# Safety and efficacy of oral DMSA therapy for children with autism spectrum disorders: Part B - Behavioral results

**DOI:** 10.1186/1472-6904-9-17

**Published:** 2009-10-23

**Authors:** James B Adams, Matthew Baral, Elizabeth Geis, Jessica Mitchell, Julie Ingram, Andrea Hensley, Irene Zappia, Sanford Newmark, Eva Gehn, Robert A Rubin, Ken Mitchell, Jeff Bradstreet, Jane El-Dahr

**Affiliations:** 1 Division of Basic Medical Sciences, Southwest College of Naturopathic Medicine, Tempe, AZ, USA; 2 Department of Pediatric Medicine, Southwest College of Naturopathic Medicine, Tempe, AZ, USA; 3 Autism Research Institute, San Diego, CA, USA; 4 Center for Integrative Pediatric Medicine, Tucson, AZ, USA; 5 Department of Mathematics, Whittier College, Whittier, CA, USA; 6 International Child Development Resource Center, Phoenix, AZ, USA; 7 Department of Pediatrics, Tulane University School of Medicine, New Orleans, LA, USA

## Abstract

**Background:**

This study investigated the effects of oral dimercapto succinic acid (DMSA) therapy on the behavioural symptoms of children with autism spectrum disorders (ASD) ages 3-8 years.

**Methods:**

Phase 1 involved 65 children with ASD who received one round of DMSA (3 days). Participants who had high urinary excretion of toxic metals were selected to continue on to phase 2. In phase 2, 49 participants were randomly assigned in a double-blind design to receive an additional 6 rounds of either DMSA or placebo.

**Results:**

The groups receiving one round and seven rounds of DMSA had significant improvements on all the assessment measures. For the seven round group, the degree of improvement on the assessment measures could be partially explained by a regression analysis based on excretion of toxic metals and changes in glutathione (adjusted R^2 ^of 0.28-0.75, p < 0.02 in all cases). One round of DMSA had nearly the same benefit as seven rounds. The assessment measures correlated reasonably with one another at the beginning of the study (r = 0.60-0.87) and even better at the end of the study (r = 0.63-0.94).

**Conclusion:**

Overall, both one and seven rounds of DMSA therapy seems to be reasonably safe in children with ASD who have high urinary excretion of toxic metals, and possibly helpful in reducing some of the symptoms of autism in those children.

## Background

Autism is a severe developmental disorder which involves social withdrawal, communication deficits, and stereotypic/repetitive behaviors. The cause(s) of autism are unknown, but both genetic and environmental factors have been implicated. One environmental factor that has received significant attention is the body burden of mercury, lead and other toxic metals [1-5].

The purpose of this paper is to evaluate the safety and efficacy of DMSA therapy on children with autism spectrum disorders (ASD), focusing on behavioral effects. The effects of DMSA on medical issues (urinary excretion of toxic metals, glutathione, blood count, blood chemistry) are discussed in the accompanying paper [6]. DMSA is FDA-approved for the treatment of lead poisoning in children as young as 2 years, and this study investigated its use for an off-label application, namely treating children with ASD who have evidence of significant heavy metal exposure (based on urinary excretion after DMSA challenge). DMSA preferentially binds to lead, but can also increase the excretion of several other toxic metals (including tin, bismuth, thallium, mercury, antimony, and tungsten) to a lesser extent. Another paper resulting from this study discusses the strong correlation of the initial severity of autism with the body burden of toxic metals [7].

## Methods

The methodology is primarily discussed in the accompanying paper [6]. This study was conducted with the approval of the Human Subjects Institutional Review Board of Southwest College of Naturopathic Medicine. All parents and (where possible) children signed informed consent/assent forms.

**Figure 1:**
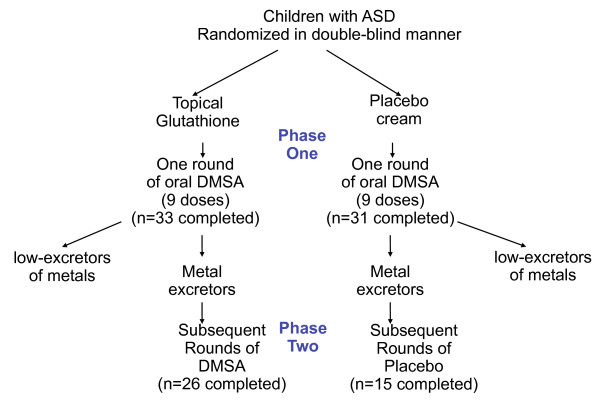
**Study design**. Both groups of participants received a single challenge round of oral DMSA; those excreting significant heavy metals continued on to Phase 2, and received additional 6 additional rounds of DMSA or placebo.

The study was designed with a screening round (nine doses over three days) of DMSA in Phase 1, followed by a randomized, double-blind, placebo-controlled study of six additional rounds (see Figure 1). The purpose of the screening round was to only allow participants with high excretion of toxic metals into phase 2. However, as discussed in the accompanying paper [6], the single screening round of DMSA had an unexpectedly dramatic effect on improving abnormal glutathione and platelet levels, and that effect lasted through the end of phase 2, so that phase 2 turned out in practice not to be a placebo-controlled investigation. Instead, it appears best to interpret this study as a comparison of the effect of one round of DMSA (and six rounds of placebo) versus seven rounds of DMSA. In discussing these two groups below, we will refer to them at the "1-round" group and the "7-round" group.

Four tools were used to assess the severity of autism, namely the Pervasive Developmental Disorder Behavior Inventory (PDD-BI) [8] Autism Treatment Evaluation Checklist (ATEC) [9], Severity of Autism Scale (SAS) [7], and the Autism Diagnostic Observation Schedule (ADOS) [10]. Different methods were used because they each assess somewhat different aspects of autism. The ATEC was evaluated at the beginning of Phase 1 and the end of phase 2, and the other three instruments were evaluated at the beginning of Phase 2 and the end of Phase 2. Also, a new instrument, the Parent Global Impressions (PGI) was used at the end of the study to evaluate changes in symptoms; it complements the other scales because is only evaluates changes in symptoms, rather than absolute levels of severity. The ATEC, SAS, PDD-BI, and PGI were assessed by the participants' parents, and the ADOS was evaluated by a certified ADOS evaluator. It should be noted that the ADOS was developed primarily for diagnosing autism, and it is unclear if it sensitive enough for treatment studies, whereas the other instruments were designed to assess severity of symptoms, not to diagnose.

### Statistical Analysis

Several types of statistical analyses were used, depending on the research question being addressed. In comparing pre/post levels within a treatment group (such as changes in a participant's autism severity), 2-sided paired t-tests were used, and for each individual hypothesis a p value of 0.05 or lower was assumed significant. Pearson correlation coefficients were obtained to determine the strengths of linear relationships among the variables involved in the analyses.

Regression analysis was employed to examine the relationship between the change in the severity of autism (assessed by the ATEC, PDD-BI, SAS, and ADOS) and the urinary excretion of toxic metals, (after the 9^th ^dose of DMSA in phase 1), and the change in glutathione (in the red blood cells). To reduce the number of variables, for the behavioral assessments we focus primarily on the summary measures (e.g, we analyzed ATEC and ADOS total scores, rather than dealing with the individual subscores comprising the total). For the selected dependent and independent variables, step-wise linear regression analyses were conducted: initially all independent variables were included in the regression; then at each step, the variable with the highest p-value was eliminated, and this process was continued until the adjusted R^2 ^value began declining. Thus, the goal was to determine the best fit to the sample data for the selected model, taking into account the correlation among the independent variables. Since the data had several missing values (due to missing lab or behavioral data), the regression analyses were conducted in two slightly different ways which generally yielded very similar results: 1) eliminate all participants with missing data for any of the variables in the model at the beginning of the analysis, and 2) eliminate participants on an as-needed basis (i.e., only where there is missing data for any variable in the current step in the analysis). Since these two methods yielded very similar results, for brevity we only report the results for method 1.

## Results

### ATEC

Table 1 summarizes the ATEC results for the participants who finished Phase 2. Note that some parents did not complete Section IV of Health/Physical Behavior due to a clerical error. For the total ATEC score, each group had a statistically significant improvement, with the 7-round group improving more (-26%) than the 1-round group (-19%), but the difference in improvement between groups was not statistically significant. The 7-round group improved more than the 1-round group on the Speech subscale and the Health/Physical Behavior subscales, but the differences were not statistically significant. Both groups improved similar amounts on the Sociability and Sensory/Cognitive Awareness subscales.

**Table 1:** ATEC scores at beginning at beginning of phase 1 and end of phase 2, for those who completed phase 2.

		**Initial**	**St dev**	**Final**	**St dev**	**% change**
**I. SPLC**	7 rounds	13.4	7.7	10.6	7.0	-21%***
	1 round	12.0	8.4	10.5	8.9	-13%
**II. Sociability**	7 rounds	16.6	8.5	12.1	6.5	-27%***
	1 round	14.9	6.8	11.2	6.5	-25%*
**III. Sensory/Cognitive Awareness**	7 rounds	16.0	7.4	11.7	7.9	-27%***
	1 round	13.0	6.8	9.6	5.9	-26%*
**IV. Health/Physical/Behavior**	7 rounds	29.9	14.4	21.6	10.1	-28%**
	1 round	21.5	9.4	18.3	9.2	-15%
**ATEC Total**	7 rounds	80.8	24.0	60.14	27.9	-26%***
	1 round	66.2	20.7	53.4	23.5	-19%**

* P < 0.05

**P < 0.01

*** P < 0.001

For the 7-round group n = 25 (n = 14 for Section IV & Total), and for the 1-round group n = 15 (n = 10 for Section IV & Total). The statistical significance of the change is indicated, based on a paired t-test.

Because of concerns about the possible side-effects of DMSA on gut function, two of the questions on the ATEC which relate to gut function were analyzed. Those questions (on constipation and diarrhea) have a severity scale of 0 = none, 1 = mild, 2 = moderate, 3 = severe. For the participants who finished phase 2, there was little change in severity of diarrhea (0.52 +/-0.79 at beginning, 0.57 +/- 0.79 at end) and a small increase in severity of constipation (0.61 +/- 0.89 to 0.87 +/- 1.1, not significant).

### PDD-BI

Table 2 lists the results for the PDD-BI. The PDD-BI is split into 2 sections, one for Maladaptive Behaviors (7 subscales) and one for Adaptive Behaviors (3 subscales). One of the Maladaptive subscales, the Semantic/Pragmatic Problems (SPP), was difficult to interpret, since some children initially had no spoken language, and then began speaking with simple words or phrases during the study; this results in a worsening of their SPP score when their language is actually beginning. Therefore, we chose not to report the results of this subscale, and not to include it in the composite scores. This modified Autism Composite score was discussed with I. Cohen, the developer of the PDD-BI.

**Table 2:** Results of PDD-BI evaluations by Parents N = 25 for the 7-round group, and N = 15 for the 1-round group.

		**Initial**	**St. Dev**	**Final**	**St. Dev**	**% Change**
**Maladaptive Behaviors**						
Sensory/Perceptual Approach Behaviors	7 Rounds	22.8	14.7	17.8	13.9	-22%*
	1 Round	20.0	13.6	13.7	13.4	-31%**
Ritualisms/Resistance to Change	7 Rounds	13.9	10.5	10.0	7.8	-28%**
	1 Round	15.0	8.5	11.5	8.2	-23%**
Arousal Regulation Problems						
	7 Rounds	17.9	9.0	13.9	8.5	-22%**
	1 Round	14.9	6.6	12.1	8.8	-19%
Specific Fears						
	7 Rounds	21.7	16.0	14.6	12.1	-33%**
	1 Round	18.7	10.3	15.9	11.1	-15%
Aggressiveness						
	7 Rounds	13.4	9.8	9.8	6.7	-27%*
	1 Round	11.4	8.1	8.4	7.2	-26%*
Social Pragmatic Problems						
	7 Rounds	16.9	9.2	14.5	9.2	-14%
	1 Round	13.9	7.5	9.9	7.5	-29%**
Semantic/Pragmatic Problems						
	7 Rounds	15.2	7.6	12.5	7.3	-18%
	1 Round	11.1	5.6	9.6	4.5	-13%
**AWP/C Composite Sensory+Ritual+SocPrag+Semantic+ Arousal+Fears+Agg**						
	7 Rounds	105.	58.6	79.5	49.9	-24%***
	1 Round	93.8	29.5	71.6	38.8	-24%***
**Adaptive Behaviors:**						
Social Approach Behaviors						
	7 Rounds	63.8	20.6	70.8	23.6	11%**
	1 Round	68.2	25.4	72.6	20.2	6%
Express (Phonological & Semantic Pragmatic)						
	7 Rounds	41.4	28.2	43.6	27.1	+5%
	1 Round	44.0	34.9	51.3	31.5	+17%*
Learning, Memory, and Receptive Language						
	7 Rounds	22.7	8.1	25.4	8.7	12%*
	1 Round	23.2	10.1	26.5	8.9	14%**
**REXSCA/C Composite SocialApp+Express+LMRL**						
	7 Rounds	127	52.7	139	54.5	9%**
	1 Round	135	65.5	150	53.6	11%*
**Autism Composite (Sensory, Ritual & Social Problems - Social Approach Behaviors - Expressive Language)**						
	7 Rounds	-53.1	66.4	-73.3	67.9	38%**
	1 Round	-63.5	65.1	-88.8	58.3	40%**

* P < 0.05

** P < 0.01

*** P < 0.001

The statistical significance of the change is indicated, based on a paired ttest.

For the modified composite score for the Maladaptive section (Sensory/Perceptual Approach, Ritualisms/Resistance to Change, Social Pragmatic Problems, Arousal Regulation, Specific Fears, and Aggressiveness), there was a similar improvement in both the 7-round and 1-round groups of 24%. For the composite score for the Adaptive Behaviors (Social Approach Behaviors, Phonological and Semantic Pragmatic, Learning, Memory, and Receptive Language) there were similar improvements in the 7-round and 1-round groups (9% and 11%, respectively). For the modified Autism composite of Maladaptive and Adaptive behaviors (Sensory/Perceptual Approach, Ritualisms/Resistance to Change, Social Pragmatic Problems, Social Approach Behaviors, Phonological and Semantic Pragmatic), there were large and similar improvements in both the 7-round and 1-round groups (38% and 40%, respectively). Overall, there were very similar improvements for the 7-round and 1-round groups, and the improvements on the Autism composite score was highly statistically significant.

### Severity of Autism Scale (SAS)

Table 3 summarizes the results for the SAS. Both the 7-round and 1-round groups improved similar amounts (18-19% reduction of their SAS scores), and the improvements were statistically significant.

**Table 3:** Scores of Autism Severity Scale by parents at the beginning of Phase 2 and the end of Phase 2.

	**Initial**	**St dev**	**Final**	**St dev**	**% change**
**7 Rounds**	5.2	2.2	4.2	2.0	-19%***
**1 Round**	5.5	2.7	4.5	2.6	-18%**

* P < 0.05

** P < 0.01

*** P < 0.001

N = 22 for the 7-round treatment group and N = 14 for the 1-round group. The statistical significance of the change is indicated, based on a paired ttest.

### ADOS

Table 4 summarizes the results for the ADOS. At the beginning of the study, based on the combined score of the Communication + Sociability scores, 81% of the participants met the criteria for autism, 12% met the criteria for ASD, and 7% were below the criteria for ASD. Overall, changes in ADOS scores were less than for the other scales. The 7-round group had slightly better improvements in sociability, play, and stereotypical behavior/repetitive interests, but the differences were not statistically significant. The two groups had similar changes in their communication and communication + sociability score. The 7-round group had statistically significant improvements in their Sociability and Communication + Sociability scores, but the 1-round group did not have any statistically significant improvements.

**Table 4:** ADOS Scores at beginning and end of Phase 1 for 7-round group (n = 26) and 1-round group (n = 15).

	**Initial**	**Final**	**% change**
**Communication**			

7 Rounds	7.8	7.1	-9%
1 Round	6.7	5.9	-11%
**Sociability**			
7 Rounds	9.3	8.3	-10%**
1 Round	8.1	7.9	-2%

**Communication + Sociability**			

7 Rounds	17.0	15.4	-9%***
1 Round	14.7	13.7	-7%
**Play**			
7 Rounds	3.2	3.0	-5%
1 Round	2.8	2.8	0%
**SBRI**			
7 Rounds	3.9	3.5	-9%
1 Round	3.5	3.5	-2%

* P < 0.05

** P < 0.01

*** P < 0.001

The statistical significance of the change is indicated, based on a paired ttest.

### Parental Global Impressions (PGI)

At the end of phase 2, parents filled out a PGI questionnaire, with a scale ranging from much worse (-3) to same (zero) to much better (+3) for 10 different areas, and an Overall score. Tables 5 lists the results. In general, there was no significant difference between the 7-round group and the 1-round group. Both groups reported similar improvements in the Overall score, and in the areas of expressive language, receptive language, cognition/thinking, play skills, and eye contact. The 1-round group reported slightly more improvement in sociability, but the difference was not statistically significant. There was little change in tantrums, stools/gastrointestinal issue or sleep problems in either group. There was a slight worsening of hyperactivity in the 7-round group. It is interesting that the average scores of the two groups were very similar in each category, with a 95% correlation between them. Table 6 shows a detailed breakdown of the Overall category, for each group and for their combination.

**Table 5:** Results of "Parent Global Impression" questionnaire, administered at end of Phase 2.

	**7 rounds (n = 24)**	**1 round (n = 12)**
**Overall**	1.7	1.6
**Expressive Language**	2.0	1.6
**Receptive Language**	1.8	1.9
**Cognition/Thinking**	1.8	1.8
**Play Skills**	1.5	1.6
**Sociability**	1.3	1.8
**Eye Contact**	1.1	0.8
**Tantruming**	0.5	0.3
**Stools/Gastrointestinal**		
**Isssues**	0.0	-0.2
**Sleep**	-0.3	-0.1
**Hyperactivity**	-0.5	-0.1

Scale ranges from no change (0), slightly better/worse (+1, -1), better/worse (+2, -2), much better/worse (+3, -3). There were no statistically significant differences between the two groups. Arranged in order of degree of improvement for 7 rounds group.

**Table 6:** Results of "Parent Global Impressions" questionnaire, for the "Overall" category

**Rating**	**7-round group**	**1 round**	**Both groups combined**
**Much Better**	29%	27%	28%
**Better**	33%	27%	31%
**Slightly Better**	25%	27%	28%
**No Change**	4%	18%	8%
**Slightly Worse**	4%	0%	3%
**Worse**	4%	0%	3%

### Summary of autism severity scales

Since the improvement in scores for the 7-round and 1-round groups were similar for all the metrics, it is useful to group both groups together and analyze the average effect of treatment for the two groups. As summarized in Table 7, if we average over the 5 assessment tools, about 77% of the participants had improved autism severity scores, about 12% had no change, and about 11% had a worsening of their scores. So, although the DMSA treatment appeared to be beneficial in most cases, there was a small subset whose symptoms appeared to worsen slightly. The most common adverse effect seemed to be hyperactivity, according to the PGI, and this effect was usually temporary.

**Table 7:** Summary of changes in autism severity scores, combining both 1-round and 7-round groups

	**% Improved**	**% No Change**	**% Worsened**
**ATEC**	96%	2%	2%
**SAS**	61%	33%	6%
**ADOS (Comm. + Social)**	68%	15%	17%
**PDD-BI (modified**	75%	2%	22%
**Autism Composite)**			
**Parent Global Impression**	86%	8%	6%
**Average of all 5 assessments**	77%	12%	11%

### Correlations of Severity Scales

Table 8 shows the correlations among the initial scores, and Table 9 shows the correlations among the final scores. Initially, there is a high correlation between the ATEC and the PDD-BI (r = 0.87), and a good correlation of the SAS with the ATEC (r = 0.70) and the PDD-BI (r = 0.72). The correlation of the ADOS with the other scales is lower (r = 0.57-0.58). At the end of the study, the correlations among the final scores are slightly higher, especially for the correlations of the ADOS with the other scales (r = 0.63-0.73). It should be noted that the ADOS evaluation was done by a professional evaluator, whereas the other assessments were done by the same parent.

**Table 8:** Correlation of Initial Severity Scores (pre vs. pre)

	**ATEC**	**SAS**	**ADOS (Social + Communication)**	**PDD-BI (modified autism)**
**ATEC**	1			
**SAS**	0.70	1		
**ADOS**	0.60	0.60	1	
**PDD-BI**	0.87	0.72	0.67	1

**Table 9:** Correlation of Final Severity Scores (post vs. post)

	**ATEC**	**SAS**	**ADOS (Social + Communication)**	**PDD-BI (modified autism)**
**ATEC**	1			
**SAS**	0.74	1		
**ADOS**	0.66	0.73	1	
**PDD-BI**	0.94	0.73	0.63	1

### Correlation of Improvement in Severity Scales with Age

A correlation analysis of age with changes in the severity scales revealed a slight positive correlation for two autism severity scales (ATEC (r = 0.24), SAS (r = 0.17), neither significant) and little correlation for the other two scales. (PDD-BI (r = 0.09), ADOS (r = -0.05). This means that the older children had almost the same degree of improvement as the younger children.

### Regression Analyses of Changes in the Severity of Autism

Table 10 shows the results of stepwise linear regression analyses for changes in the various autism severity scales as a function of urinary excretion of toxic metals (after the 9th dose of DMSA in phase 1) and the change in glutathione. The 1-round and 7-round groups were combined for this analysis, with separate coefficients for each group. The prediction of PDD-BI and ATEC yielded significant results, but only the terms related to the 7-round groups were significant. For the ADOS and SAS there were no significant results. Table 10 displays the results.

**Table 10:** Regression Analyses of Changes in Autism Severity vs. Urinary Metal Excretion (after 9^th ^dose of DMSA in Phase 1) and Changes in Glutathione (Dglut).

	**Adjusted R^2^**	**P-value**	**Equation**	**Most significant variables**
**Change in PDD-BI (n = 32)**	0.59	0.002	7-rnd group (17.9 -2.62 Pb9 +0.230 Sn9 +102Tl9 -135Sb9 - 1.01 As9 -0.0695 Dglut) 1-rnd group (-32.7 -0.564 Pb9 +0.0755Sn9 +17.4 Tl9 +13.8 Sb9 -0.476 As9 +0.045 Dglut)	7-rnd group: Dglut***, As9**, Sb9**, Tl9**, Pb9*
				1-round group: No significant terms
**Change in ATEC (n = 32)**	0.25	0.02	7-rnd group (1.93 -7.82 Hg9 -0.352 As9) 1-rnd group (-4.91 -0.672 Hg9 -0.260 As9)	7-rnd group: Hg9**, As9*
				1-rnd group: No significant terms
**Change in SAS (n = 29)**	0.22	n.s.	(not listed since not significant)	
**Change in ADOS (comm.. + social) (n = 32)**	0.18	n.s.	(not listed since not significant)	

Both treatment groups analyzed together, with individual coefficients for each group. Only the PDD-BI and ATEC yielded significant results, and only the 7-round group variables had significant coefficients. In the regression equation, the suffixes for the metals refer to the value at Baseline (B) and after the 9^th ^dose of DMSA (9). Note that initial glutathione did not contribute to any of the final equations.

Since only the 7-round group had significant coefficients, all the regression analyses were redone for the 7-round group only. The results are listed in Table 11. This re-analysis yielded much better predictions (adjusted R^2 ^from 0.28 to 0.75), and all of the results were significant (p-values from 0.02 to 0.0006). This demonstrates that the behavioral changes observed in the 7-round group could be significantly described by urinary excretion of toxic metals and change in glutathione. For the PDD-BI (which had the highest adjusted R^2 ^and the most significant p-value), the major factors were excretion of thalium (Tl) and arsenic (As) and changes in RBC glutathione, followed by excretion of lead (Pb) and antimony (Sb). Overall, the most consistent significant factors between the different scales were excretion of As (ATEC, ADOS, PDD-BI), excretion of Tl (SAS, PDD-BI), excretion of Pb (SAS, PDD-BI), and change in RBC gluathione (SAS, PDD-BI). Other significant contributors were mercury (Hg) (ATEC), aluminum (Al) (ADOS), and Sb (PDD-BI). Since the toxic metal excretions exhibit considerable correlation amongst themselves [6], one should refrain from reading too much into the relationships between specific metals and severity of autism. Instead, one should interpret the results as indicating a general relationship between changes in autism severity and urinary excretion of toxic metals and change in glutathione for the 7-round group, but much less significance when combined with the 1-round group.

**Table 11:** Regression Analyses of Changes in Autism Severity vs. Urinary Metal Excretion (after 9^th ^dose of DMSA in Phase 1) and Changes in Glutathione (Dglut).

	**Adjusted R^2^**	**P-value**	**Equation**	**Most significant variables**
**Change in PDD-BI (n = 20)**	0.75	0.0006	10.25 -2.48Pb9 +0.169Sn9 +113.5Tl9 -119.1Sb9 - 1.199As9 +0.726Al9 - 0.0608DGlut	Tl9**, As9**, DGlut**, Pb9*, Sb9*
**Change in ATEC (n = 20)**	0.44	0.006	9.41 -0.663Pb9 -8.80Hg9- 0.352As9	Hg9**, As9*
**Change in SAS (n = 18)**	0.57	0.002	-1.01 +0.0985Pb9- -0.00169DGlut	Tl9**, Pb9*, DGlut*
**Change in ADOS (comm.. + social) (n = 20)**	0.28	0.02	-1.91+00.0472As9 - 0.0877Al9	As9*, Al9*

This is the same analysis as Table 10, except it is for the 7-round group only, resulting in much better regression fit and much greater significance in all cases. This demonstrates that the changes in the 7-round group are much better predicted by their excretion of heavy metals and change in glutathione.

## Discussion

### Discussion - Effect of DMSA on Symptoms of Autism

Five different tools (ATEC, SAS, PDD-BI, ADOS, PGI) were used to assess changes in the severity of autism during the course of the study. Four of the scales (ATEC, SAS, PDD-BI, and ADOS) could be analyzed for correlations, and reasonable correlations were found between them at the beginning (pre vs pre) and end of the study (post vs post), which supports the validity of each of them. The correlations were highest among the ATEC, SAS, and PDD-BI, which is expected since the same parent conducted those, whereas the ADOS was evaluated by a certified professional.

Three of the assessments (PDD-BI, SAS, and PGI) found significant but similar improvements in both the 1-round and 7-round groups. Two of the assessment tools (ATEC and ADOS) found somewhat more improvement in the 7-round group, but the difference was not statistically significant. Overall, one round of DMSA seemed to provide almost as much benefit as seven rounds of DMSA.

If phase 2 were a true placebo-controlled study, then this result would mean that the DMSA had little or no effect on behavior. However, it is important to remember two major points:

1) As discussed in the accompanying paper (Adams et al 2009), the single round of DMSA in Phase One had a dramatic effect on normalizing glutathione and improving platelet levels, and additional doses did not further improve platelet levels, and probably did not significantly affect glutathione since it was already normal or near-normal after the first round. 2) The degree of improvement correlated with excretion of toxic metals and changes in glutathione, especially for the PDD-BI, with an extremely high adjusted R^2 ^of 0.75, and a highly significant p-value of 0.0006. If the reported improvements were purely a random placebo-effect, adjusted R^2 ^values would be near zero and not significant. Thus, most of these improvements appear to be real and directly related to excretion of toxic metals and changes in glutathione.

Thus, this data suggests that the DMSA may have had a beneficial effect on the symptoms of autism. A future randomized, double-blind, placebo-controlled study without an initial screening round of DMSA would help confirm this.

The findings of this paper are consistent with our findings that the initial severity of autism significantly correlated with the body burden of toxic metals, assessed pre and post DMSA challenge [7]. It was surprising that just one round of DMSA had significant behavioral effects, but this is consistent with the finding that just one round of DMSA was sufficient to normalize glutathione and improve platelet levels (a marker of inflammation). It seems likely that a longer treatment study may yield even more beneficial results, since 80% of the children were still excreting high levels of lead and other toxic metals at the end of 7 rounds of DMSA therapy. It appears that monitoring urinary excretion of toxic metals may be a useful guide as to how long to continue therapy, especially if ongoing exposure to toxic metals is minimized.

Overall, the results of this study and others suggest that toxic metals contribute to the severity of autism, and may be a factor in causing autism in some cases. This is consistent with the general role of toxic metals as neurodevelopmental toxins. If this is the case, a reduction in exposure to toxic metals might be helpful in partially reducing the incidence of autism. More research is needed to investigate this possibility.

### Age Effects

Improvements in the various autism severity scores had a slight positive correlation with age for the ATEC and SAS, (not statistically significant, but essentially no correlation for the PDD-BI or ADOS. This suggests that age had little effect on degree of improvements, with the older children improving as much or almost as much as the younger children.

### Gut symptoms

There has been some concern that DMSA therapy may result in gut problems, possibly related to the overgrowth of yeast. However, following the protocol of this study, we did not observe any evidence of significant change in gut problems. Based on the PGI, there was no change in gut problems on average, and based on the individual questions on the ATEC, there was, on average, no change in diarrhea and only a slight worsening of constipation (not significant). Thus, it appears that oral DMSA, by itself, does not cause or significantly exacerbate gut problems in most cases. A limitation of this study is that we did not perform extensive evaluations of gut flora or gut function.

### Comparison with other treatments

It is interesting to compare the efficacy of DMSA therapy with other therapies. This is difficult because different scales are used in different treatment studies (which is part of the reason why we included several scales in this study). The PGI assessment used in this study is very similar to an earlier version of the PGI used in a 3-month double-blind, placebo-controlled vitamin/mineral treatment study [11]. DMSA therapy appeared to provide similar improvements to vitamin/mineral therapy in the areas of Expressive Language, Receptive Language, Sociability, and Overall. However, the vitamin/mineral therapy yielded better improvements in sleep and gastrointestinal problems, and possibly slightly better eye contact.

## Conclusion

1) The severity of autism, as assessed by five different assessment tools, significantly decreased during the study for both the group receiving 1 round of DSMA (and 6 rounds placebo) and 7 rounds of DMSA.

2) Regression analysis of improvement in autistic symptoms with glutathione and metal excretion (especially thallium, arsenic, mercury, and lead) suggests most of the improvement was real (not due to a placebo effect), and thus suggests DMSA may have resulted in reduction of some symptoms of autism.

3) Age had little effect on the degree of improvement; older children improved as much or almost as much as the younger children on the various scales.

4) Overall, DMSA therapy appears to be generally safe, and possibly effective in reducing the symptoms of autism in some children.

Future studies should consider a double-blind, placebo-controlled design (no screening round) to definitively determine the effect of a single round of DMSA on behavior. Also, a longer term treatment study (longer than 7 rounds) should be considered to determine if longer treatment results in greater benefits.

## Competing interests

The authors declare that they have no competing interests.

## Authors' contributions

JBA was the primary organizer of the study, did the initial data analysis, and was the primary author. He was the official co-principal investigator for the study. MB was the primary physician for the greater Phoenix area. He was the official principal investigator, and worked with JBA on study design and study approval. EG was the study coordinator and lead study nurse. JM was a secondary physician who assisted MB. JI was the ADOS evaluator for the greater Phoenix area. AH was a study nurse. IZ was the ADOS evaluator for the greater Tucson area. SN was the physician for the greater Tucson area. EG handled data entry and helped with data analysis. RAR was the primary statistician for the study, and did the regression analysis. KM was the pharmacist who assisted with compounding the DMSA. JB was a physician who served as consultant for the study. JME was a physician who served as a consultant for the study. All authors read and approved of the final manuscript.

## Pre-publication history

The pre-publication history for this paper can be accessed here:


